# Development of machine learning predictive model for type 2 diabetic retinopathy using the triglyceride-glucose index explained by SHAP method

**DOI:** 10.3389/fendo.2025.1631647

**Published:** 2025-11-10

**Authors:** Xiaoqin Liu, Shuying Wu, Yue Yang, Yang Li, Xinting Zhang, Rihui Liu, Ling Qin, Fei Li

**Affiliations:** 1 The First Hospital of Jilin University, Changchun, Jilin, China; 2 Guangdong Mental Health Center, Guangdong Provincial People's Hospital (Guangdong Academy of Medical Sciences), Southern Medical University, Guangzhou, Guangdong, China; 3 Meihekou Central Hospital, Meihekou, Jilin, China

**Keywords:** TyG-index, diabetic retinopathy, machine learning, predictive model, SHAP

## Abstract

**Introduction:**

This study aimed to develop a diabetic retinopathy (DR) Prediction model using various machine learning algorithms incorporating the novel predictor Triglyceride-glucose index (TyG). Furthermore, the model was interpreted using the SHapley Additive exPlanations (SHAP) method.

**Method:**

Real-world data were collected from a general hospital in a major city and a county clinic, then divided into the DR Group (1392) and non-DR group (2358). Baseline data were collected, and variables were selected using Recursive Feature Elimination with Cross-Validation (RFECV). The performance of five machine learning algorithms, including Logistic Regression model (LR), Decision Tree (DT), Random Forest (RF), Support Vector Machine (SVM), and XGBoost (XGB), was assessed based on accuracy, sensitivity, specificity, and Area Under the Curve (AUC) of the Receiver Operating characteristic Curve (ROC). The optimal model was interpreted using SHAP.

**Result:**

LVM and LR demonstrated superior performance in both the test set and training set (ROC, 0.85 and 0.82, respectively). The top five predictors identified by SHAP analysis included TyG, Insulin therapy, HbA1c, Diabetes Course, HDL. HDL was identified as a protective factor, while the remaining factors were associated with retinopathy.

**Conclusion:**

LR and SVM demonstrated the best performance. To our knowledge, this is the first machine learning-based DR prediction model integrating the triglyceride-glucose index (TyG) as a core predictor, overcoming limitations of insulin resistance (IR) assessment in resource-limited settings. TyG provides a cost-effective alternative to conventional IR biomarkers (e.g., HOMA-IR), enabling practical DR risk stratification in primary care.

## 1 Introduction

Diabetes has a high incidence and mortality rate, thus emerging as a major global health challenge. The global prevalence of diabetes in people aged 20~79 years may increase to 12.2% by 2045 (1). Diabetic retinopathy (DR), the most common and serious ocular complication of type 2 diabetes, can result in visual impairment and even permanent vision loss (2). To date, DR is the leading cause of blindness among individuals aged 20~74, contributing to new cases of preventable blindness in developing countries (3, 4). A meta-analysis showed that the worldwide prevalence of DR is about 22.27% (5). Notably, China has the largest number of diabetic patients in the world (1), with about 27.9% of diabetes cases being DR (6), of which 34% are in rural areas. Most DR patients are asymptomatic or mild in the early stage and are only detected when great damage has occurred or at the late stage (2). Therefore, early screening of DR is crucial. However, the detection rate of DR remains suboptimal (7), particularly in certain primary hospitals and remote regions due to the constraints in medical conditions and limited social resources. Therefore, more accessible methods are needed to aid healthcare professionals in diagnosis and screening of DR.

To date, factors such as diabetes duration, age, BMI, smoking, blood pressure, HbA1c levels, and cholesterol have been identified as risk factors for diabetic retinopathy (DR) (8, 9). However, only a limited number of studies have incorporated insulin resistance (IR) into DR prediction models, despite substantial evidence (10, 11)demonstrating a strong association between IR and DR. The hyperinsulinemic-euglycemic clamp (HIEC) is the gold standard for detecting IR (12), However, it is expensive and complex, limiting its use. HOMA-IR (13) is the most commonly utilized method for estimating insulin resistance; however, it requires the measurement of fasting insulin levels, which imposes specific technical demands on laboratory capabilities. Additionally, this method is not suitable for patients undergoing insulin therapy and is not widely adopted in primary care settings or resource-limited regions. Therefore, HOMA-IR cannot be widely promoted in primary hospitals and poor areas (14, 15). A new index has been recently identified for detecting IR: Triglyceride-glucose index (TyG) (16), TyG is calculated by fasting triglyceride (TG) and fasting blood glucose (FBG), providing a simple, reliable, and cheap detection tool (17). Besides, this index tool has shown better results than HOMA-IR (18), Elsewhere, Srinivasan (19) and Yao (20) have shown that TyG and DR are closely related, while others not.

In recent years, artificial intelligence (AI) has demonstrated significant advantages in disease prediction. Machine learning (ML), encompassing both deep learning (DL) and traditional ML approaches represents the most widely adopted technique (21). While DL has proven highly effective in medical image analysis—as evidenced by studies on brain tumor classification using DCNN (22), U-Net-based MRI segmentation (23), and mammography interpretation *via* XAI-RACapsNet (24)—its superior performance is primarily confined to high-dimensional (imaging) data. Furthermore, DL imposes higher computational demands than traditional ML methods. Due to the inherent opacity of its multiple processing layers (the so-called "black box" problem), DL poses greater challenges to model interpretability and accountability (25). In contrast, traditional ML algorithms (e.g., SVM, LR) exhibit distinct advantages when processing tabular data: superior interpretability through SHAP, critical for clinical trust (26); reduced computational demands, enabling deployment in resource-limited settings (27); and comparable performance to DL on tabular biomedical data with limited samples (27). Given our goal of developing a primary-care-suitable tool, we prioritized lightweight, interpretable models over complex DL architectures. In the absence of imaging inputs, ML outperforms DL in tabular data prediction. The newly discovered predictors can improve the predictive value for the prediction model (28, 29), TyG, as a new index for measuring IR, has shown great value in many studies, While integrating the TyG as a novel predictor further enhances accessibility without compromising performance. However, no prediction model uses TyG to predict the DR among the DM2 population.

To bridge this critical gap, we present the first machine learning framework integrating TyG for DR prediction, which simultaneously addresses three key limitations of prior approaches: 1) Methodological Barrier: Existing models (30–33) neglect insulin resistance (IR) or rely on impractical biomarkers [e.g., HOMA-IR requiring fasting insulin (11)]; 2) Clinical Applicability: TyG leverages routine lipid/glucose tests, enabling IR assessment in resource-limited settings where specialized assays are unavailable (17, 18); 3) Model Interpretability: We employ SHAP to decode the "black box" nature of ML models, quantifying TyG's non-linear contribution to DR risk. By validating this approach across multi-tier healthcare centers (urban tertiary vs. rural primary hospitals), we establish TyG as a novel, deployable biomarker for scalable DR screening.

## 2 Methods

### 2.1 Research design

#### 2.1.1 Population

This is a retrospective study, where patient data were extracted from the real-world database of The First Hospital of Jilin University (a general hospital in a major city) and Meihekou Central Hospital (a primary health care institution in the county) from January 1, 2010 to December 31, 2023.

#### 2.1.2 Inclusion criteria

a. Patients diagnosed with type 2 diabetes(T2DM) following the criteria of the 2024 American Diabetes Association (34); b. Patients aged≥18 years; c. Patients with complete indicators (triglyceride and fasting glucose).

#### 2.1.3 Exclusion criteria

a. Patients diagnosed with retinopathy at admission; b. Patients suffering from other retinal diseases, glaucoma, optic neuropathy, or eye diseases caused by systemic diseases; c. Patients with a history of eye surgery; d. Patients with severe systemic diseases (cancer, myocardial infarction, and dialysis history); e. Patients with data loss exceeding 20%.

#### 2.1.4 Outcome

The extraction variable was the first measurement record at first admission. Diagnosis information was extracted from the discharge diagnosis, and the follow-up was conducted until the first DR diagnosis, otherwise the last visit time was selected as the follow-up endpoint. Diagnostic criteria of DR included: A spectrum of retinal microvascular lesions on retinal examination with diabetic patients (35), including mild, moderate, and severe non-proliferative DR and proliferative DR. DR was diagnosed using 45° photos of macular center and indirect ophthalmoscopy when pupils were dilated. DR diagnosis was mainly achieved by endocrinologists and ophthalmologists. Patients diagnosed with retinopathy in other hospitals during the follow-up process were marked as DR, and the follow-up time was based on the earliest diagnosis time. Positive and negative samples were defined as DR and non-DR patients, respectively.

#### 2.1.5 Ethical approval

This study was conducted following the Helsinki Declaration and was approved by the Research Ethics Committee of The First Hospital of Jilin University (approval number: 2024-918). Each participant provided signed written informed consent. This study was reported based on TRIPOD (28).

#### 2.1.6 Baseline data collection

Data indicators, including sex, age, height, weight, smoking, drinking, course of T2DM, insulin therapy, hypertension history, and laboratory parameters, were mainly obtained from literature reports (2, 36, 37).

Laboratory parameters included glycated hemoglobin (HbA1c, g/dL), total cholesterol (TC, mmol/L), high-density lipoprotein (HDL, mmol/L), low-density lipoprotein (LDL, mmol/L), triglyceride (TG, mg/dL), fasting blood glucose (FPG, mg/dL), fasting C-Peptide (C-PE, ng/ml), fasting insulin (FINS,μU/ml) and C-reactive protein (CRP, μg/L).

Body mass index (BMI) was was calculated using the formula as follows: weight (kg)/ height (m)^2^. TyG index: LN [triglyceride (mg/dl)×plasma glucose (mg/dl)/2]. Three graduate students collated and cross-checked the collected data. A unified training for the data collection and collation personnel was conducted to ensure the accuracy and consistency of the data.

#### 2.1.7 Sample size calculation

The sample size was mainly based on Riley (38) standard for accurate estimation. Four sample sizes (at least 878) (predicted value with small average error, intercept model only, ensuring shrinkage coefficient of 0.9, and ensuring small optimism of apparent model) were calculated. The total sample size was at least 966 [878 + 10% (878)], considering that some medical record information was incomplete and 10% contingency.

#### 2.1.8 Model construction

Three single model algorithms (LR, DT, and SVM) and two integrated methods (RF and XGBoost) were used to train the model. LR is a machine learning method for solving binary classification problems, and is used to estimate the possibility of something. DT is a simple and easy-to-operate tree-type classification prediction model, providing intuitive and easy-to-understand results. However, DT can easily lead to overfitting during the classification process. SVM is widely used to construct a hyperplane concept to classify the observed values and can be used to deal with classification and regression problems. Compared with the single DT model, the integrated algorithms have higher accuracy but present more complicated and difficult results to explain.

#### 2.1.9 Statistical analysis

##### 2.1.9.1 Statistical interpretation

R4.2.1 software was used for all data analysis. The variables missing more than 20%, including fasting C-Peptide, fasting insulin, and CRP were deleted to improve the utilization rate of the data. For individual missing values, the average interpolation method and mode interpolation method were used for counting data and measuring data, respectively. The normally distributed data were expressed as X ± S, and compared using two independent samples T-test. The non-normally distributed data were expressed as P50 (P25, P75) and compared using Mann-Whitney U test. The counting data were expressed as frequency (%) and compared using X^2^ test. The correlation between the predictive indicators and retinopathy was assessed using univariate and multivariate logistic regression models. Variables with univariate analysis *P* < .05 were included in multivariate logistic regression analysis. The odds ratio (OR) and corresponding 95% confidence interval (CI) were used to indicate the trend of correlation.

##### 2.1.9.2 Variable selection

The model was trained and verified using Python3.9.15 software and tool kits Sklearn1.0.2, XGBoost1.7.4, and shap0.41.0. The recursive feature elimination with cross-validation (RFECV) was used to screen the optimal index. The least important features were continuously eliminated by training the model. The performance of the model was evaluated until the optimal performance index was reached. In this study, the variables with P < 0.1 in the comparison between groups were included in the RFECV model, and the best indicators were selected for subsequent prediction.

##### 2.1.9.3 Model performance assessment

The prediction efficiency of LR, DT, SVM, RF, and XGBoost 5 models was evaluated based on accuracy, sensitivity, specificity, and Area Under the Curve (AUC) of receiver operating characteristic curve. Accuracy, precision, recall rate, and F1 score were used as the indicators. SHAP was used to visually explain the machine learning model. The Summary Plot, Shap Heat map, and importance ranking diagram were also drawn. The actual application of a single sample model was visualized *via* Force Plot. *P* < .05 was considered a statistically significant difference.

To further assess model stability, we performed 5-fold cross-validation on the entire dataset. Performance metrics (AUC, accuracy, sensitivity, specificity) were averaged over 5 iterations with standard deviations calculated.

##### 2.1.9.4 Model interpretation

We employed SHapley Additive exPlanations (SHAP) (26), to interpret model predictions using Python's shap package (v0.41.0). Given computational advantages for tree-based models, SHAP analysis was applied to the XGBoost classifier *via* TreeExplainer. SHAP values – quantifying each feature's marginal contribution to predictions – were calculated for every sample in the training dataset. Four interpretability visualizations were generated: Global Feature Importance: Features ranked by mean absolute SHAP value. Summary Plot: Dot-plot distribution of SHAP values per feature. Horizontal position indicates impact direction (positive/negative SHAP); color encodes feature value (red=high, blue=low). Force Plot: Illustrates additive feature contributions driving an individual prediction from the base value (dataset average) to the model output f(x). Partial Dependence Plot: Generated using scikit-learn’s PartialDependenceDisplay, depicting the marginal effect of TyG on predicted DR risk while averaging other features.

##### 2.1.9.5 Multicollinearity assessment

To evaluate potential multicollinearity among predictors, we calculated the variance inflation factor (VIF) for all variables included in both the machine learning models and multivariate logistic regression. VIF values < 5 were considered indicative of no significant multicollinearity.

## 3 Result

### 3.1 Social demographic and clinical characteristics

**Figure 1:**
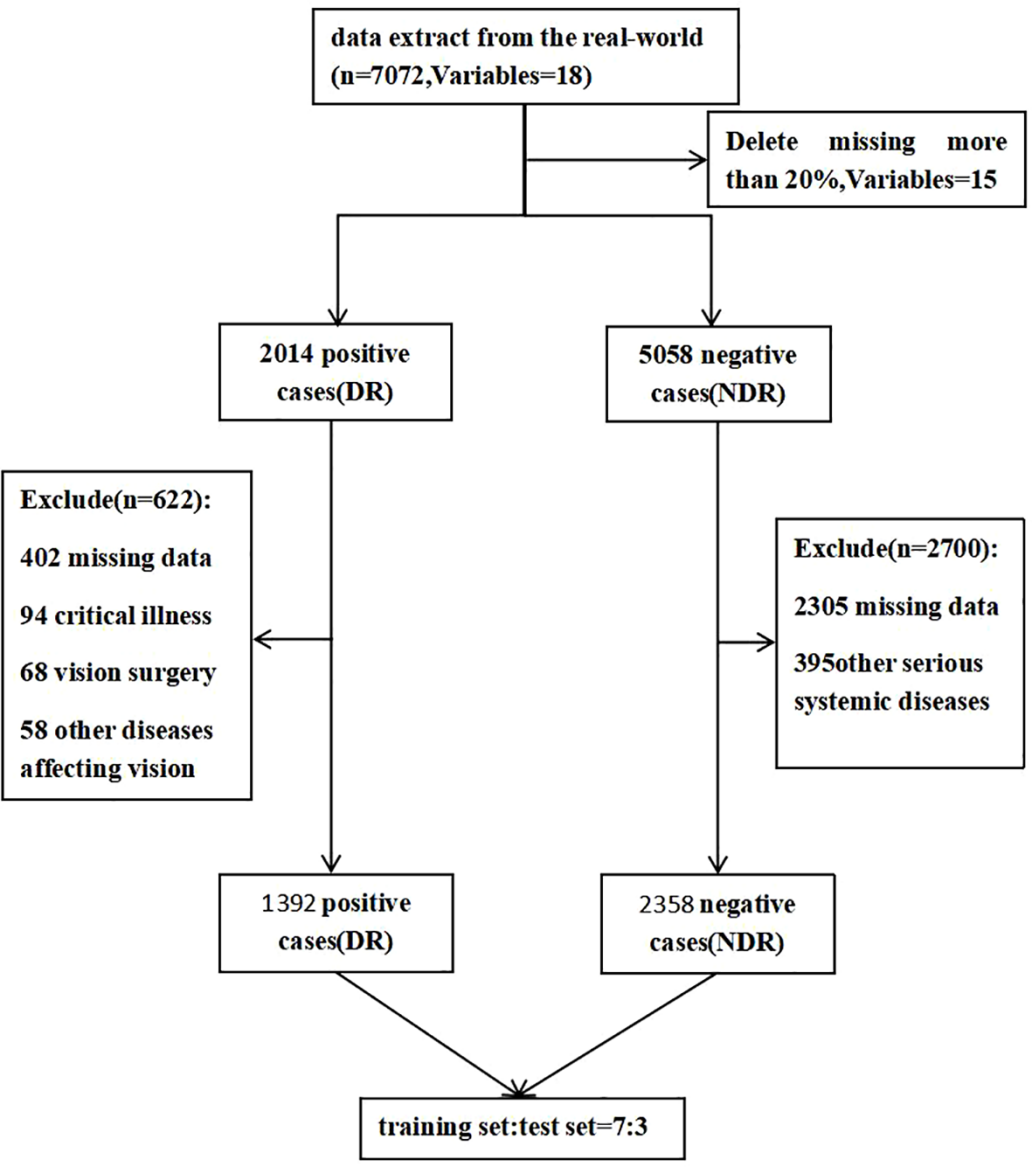
Flowchart of participant selection. DR, diabetic retinopathy; NDR, No DR.

The distribution of study participants is shown in **Figure 1**. A total of 2014 positive cases were extracted from real-world data, and 5058 negative cases were extracted *via* the computer random sampling method. Finally, 622 cases were excluded from the positive group (including 402 with missing data, 108 with other diseases affecting vision, 108 with vision surgery, and 94 with critical illness) and 2700 cases were excluded from the negative group (2305 with missing data and 395 with other serious systemic diseases), leaving 3755 cases (1392 cases in the positive group and 2358 cases in the negative group) About 63.09% of the remaining cases were males and 36.91% were females. The subjects were aged 18~91 years, with an average age of 54.8 ± 12.3 (**Table 1**).

**Table 1:** Comparison of baseline data between the two groups.

Variables		Overall	Control	Case	P
		(n=3750)	(n=2358)	(n=1392)	
Sex	Female	1384 (36.91)	800 (33.93)	584 (41.95)	<.001
	Male	2366 (63.09)	1558 (66.07)	808 (58.05)	
Age		54.8 ± 12.3	53.3 ± 12.8	57.2 ± 11.1	<.001
BMI		25.9 ± 3.7	26.0 ± 3.8	25.7 ± 3.4	.021
Smoking	No	2798 (74.61)	1724 (73.11)	1074 (77.16)	.007
	Yes	952 (25.39)	634 (26.89)	318 (22.84)	
Drinking	No	2912 (77.65)	1803 (76.46)	1109 (79.67)	.025
	Yes	838 (22.35)	555 (23.54)	283 (20.33)	
Hypertension	No	1922 (51.25)	1313 (55.68)	609 (43.75)	<.001
	Yes	1828 (48.75)	1045 (44.32)	783 (56.25)	
Insulin therapy	No	1484 (39.57)	1248 (52.93)	236 (16.95)	<.001
	Yes	2266 (60.43)	1110 (47.07)	1156 (83.05)	
Course of diabetes		10.0 (4.0, 15.0)	6.0 (3.0, 10.1)	12.0 (8.0, 20.0)	<.001
HbA1c		8.7 (7.3, 10.2)	8.2 (7.0, 9.8)	9.3 (8.2, 10.7)	<.001
TC		4.9 (4.2, 5.7)	4.9 (4.3, 5.7)	4.8 (4.1, 5.6)	.001
HDL		1.1 (1.1, 1.3)	1.1 (1.1, 1.2)	1.1 (0.9, 1.3)	<.001
LDL		3.0 (2.4, 3.5)	3.0 (2.5, 3.6)	2.9 (2.3, 3.4)	<.001
TG		2.0 (1.3, 3.4)	2.0 (1.3, 3.2)	2.0 (1.4, 4.0)	.072
FBG		8.9 (6.9, 12.0)	8.5 (6.7, 11.6)	9.7 (7.3, 12.6)	<.001
TyG		2.2 (1.7, 2.9)	2.2 (1.6, 2.8)	2.3 (1.7, 3.0)	<.001

The variables with univariate P<0.05 were included in multivariate logistics regression analysis. The results showed that HDL (*P*<.001, OR = 0.176, 95% CI:0.144~0.215), smoking (*P* = .002, OR = 0.759, 95% CI:0.620~0.92), LDL (*P* = .021, OR = 0.811, 95% CI:0.678~0.969), BMI (*P* = .001, OR = 0.815, 95% CI:0.726~0.915), TyG (*P* = .038, OR = 1.091, 95% CI:1.005, 1.185), age (*P* = .014, OR = 1.149, 95% CI:1.028~1.284), hypertension history (*P*<.001, OR = 1.506, 95% CI:1.273~1.781), course of T2DM (*P*<.001, OR = 1.798, 95% CI:1.615~2.002), insulin therapy (*P*<.001, OR = 3.166, 95% CI:2.621~3.824), HbA1c (*P*<.001, OR = 3.443, 95% CI:2.879~4.116), were the independent influencing factors of DR (**Table 2**).

**Table 2:** Results of multivariate logistic regression analysis.

Variable	B	Wald	P	OR (95%CI)
HDL	-1.736	291.731	<.001	0.176(0.144, 0.215)
Smoking	-0.275	7.136	.008	0.759(0.620, 0.929)
LDL	-0.21	5.3	.021	0.811(0.678, 0.969)
BMI	-0.205	12.035	.001	0.815(0.726, 0.915)
TyG	0.087	4.319	.038	1.091(1.005, 1.185)
Age	0.139	6.005	.014	1.149(1.028, 1.284)
Hypertension	0.409	22.879	<.001	1.506(1.273, 1.781)
Course of diabetes	0.587	115.088	<.001	1.798(1.615, 2.002)
Insulin_therapy	1.152	143.06	<.001	3.166(2.621, 3.824)
HbA1c	1.236	183.72	<.001	3.443(2.879, 4.116)

### 3.2 RFECV screening

**Figure 2:**
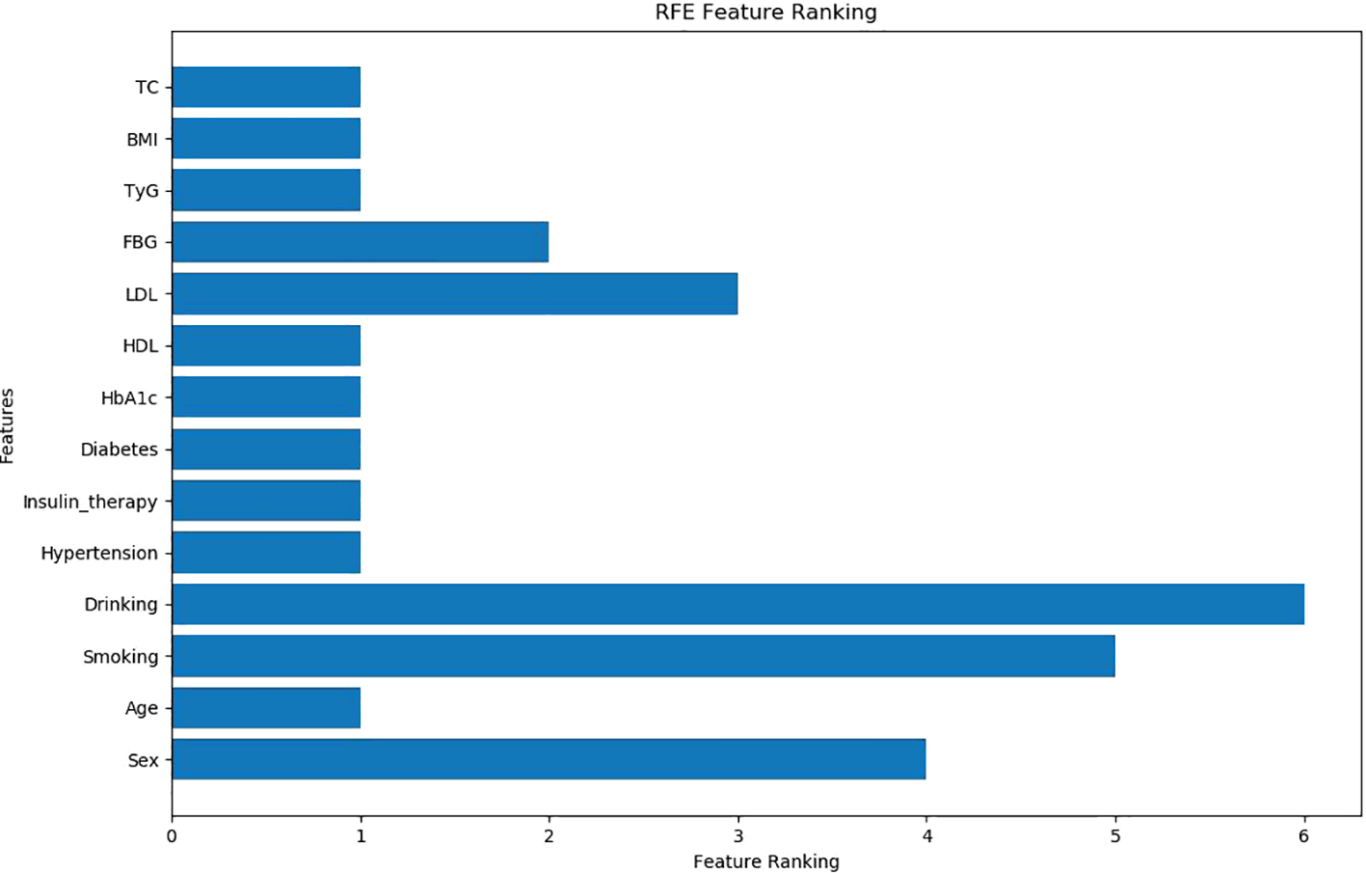
Selection results of RFECV method (9 indexes were retained, 5 indexes were excluded).

The independent variables were selected *via* RFECV method, and recursive features were eliminated. The results showed that nine indexes, including age, BMI, Diabetes course, Insulin therapy, Hypertension, HbA1c, TC, HDL, and TyG, were retained, and five indexes (sex, smoking, drinking, FBG, and LDL) were excluded (**Figure 2**).

### 3.3 Training and verification of the model

**Figure 3:**
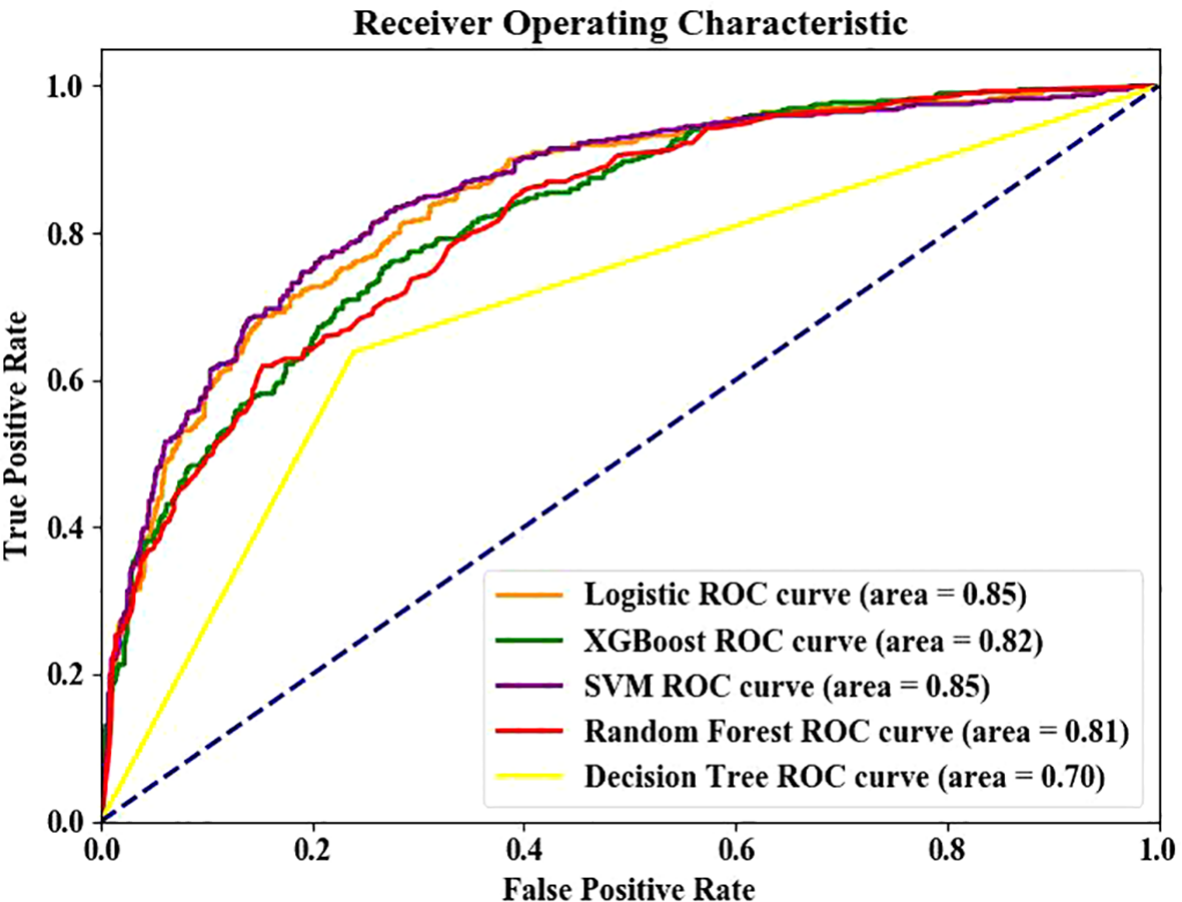
Receiver operating characteristic (ROC) curves based of five machine learning models.

The data were grouped into the training set and testing set (7:3). The last nine indexes were used as the optimal solution for model training. Moreover, SVM and LR showed the best performance in the test set and training set. The AUC curves of the five models are shown in **Figure 3**. The comparison of various prediction indexes (**Table 3**).

**Table 3:** Prediction performance indicators of five models in the training and testing sets.

Model	Train set				Test set			
	Precision	Recall	F1-score	Accuracy	Precision	Recall	F1-score	Accuracy
LR	0.70	0.58	0.64	0.75	0.70	0.69	0.70	0.79
XGBoost	0.69	0.62	0.65	0.75	0.65	0.62	0.64	0.75
SVM	0.70	0.60	0.65	0.75	0.73	0.67	0.7	0.80
RF	0.68	0.61	0.64	0.74	0.64	0.64	0.64	0.75
DT	0.59	0.56	0.58	0.69	0.59	0.64	0.62	0.72

Five-fold cross-validation confirmed model stability: Logistic Regression: AUC 0.824 ± 0.012, Accuracy 0.760 ± 0.007; SVM: AUC 0.815 ± 0.007, Accuracy 0.762 ± 0.016; The minimal standard deviations (<0.02) indicate robust performance across data subsets.

### 3.4 SHAP model analysis

#### 3.4.1 Ranking of feature importance

**Figure 4:**
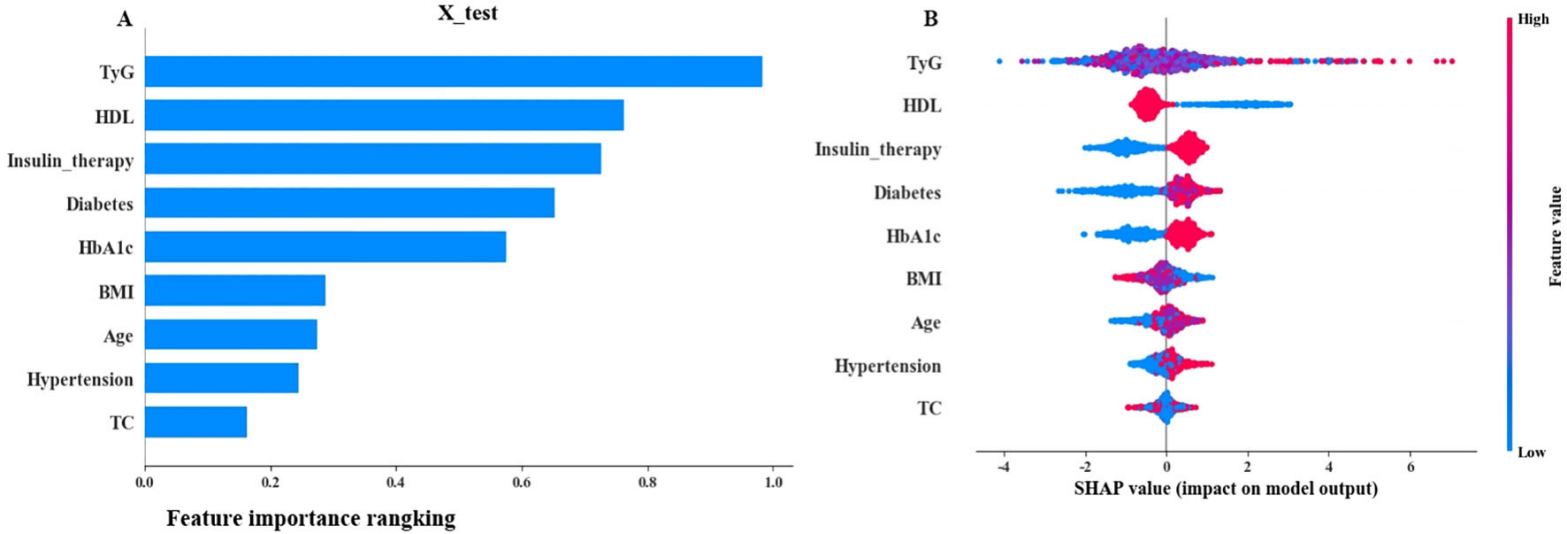
SHAP interpretation **(A)** The importance ranking of the model prediction features. The horizontal coordinate represents the SHAP values. The larger SHAP values indicate that the variable is more important; **(B)** Each point represents a feature value, and different colors represent the final influence of the feature on the model output results. Red and blue represent larger and smaller values, respectively.

The top 5 variables based on SHAP values in the ML model are shown in **Figure 4**. A summary plot is shown in **Figure 4B**, where “red” and “blue” represent higher and lower eigenvalues, respectively. SHAP value<0 indicates negative influence, SHAP value>0 indicates positive influence. The dispersion of feature distribution (located above the Y axis) was directly related to the importance of the feature. Furthermore, TyG, Insulin therapy, HbA1c, and Diabetes course showed positive effects on retinopathy, while HDL showed negative effects.

#### 3.4.2 Practical application of the model

**Figure 5:**
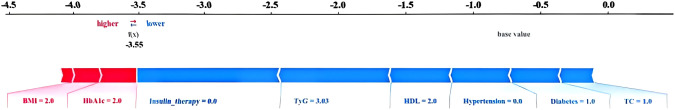
Force plot of SHAP analysis method. NDR patient.

The force Plot is shown in **Figure 5**. The blue and red arrows indicate that this factor reduces and increases the risk of retinopathy, respectively. The reference value represents the average SHAP value of all samples. F(x) represents the comprehensive SHAP value of each patient. The model can only predict the patient's retinopathy if the value of f(x) is greater than the base value. The 1045th case (**Figure 5**) was randomly predicted using the test set. Notably, f(x) was less than the base value, which was accurately predicted as the control group.

#### 3.4.3 The effect of key features on outcomes

**Figure 6:**
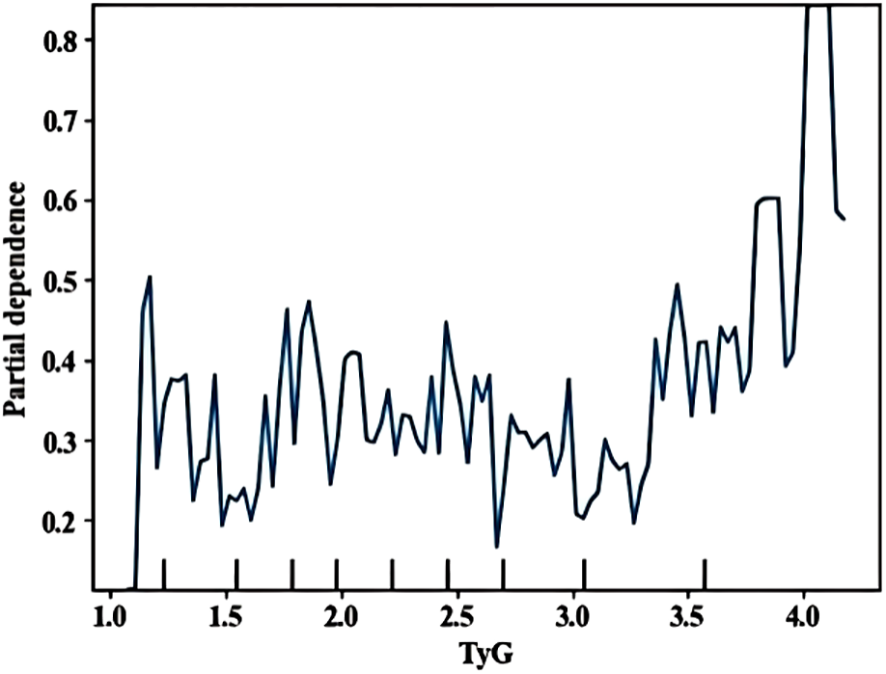
Partial dependence plots of SHAP analysis method (TyG).

A partial dependence diagram of the influence of the first three indicators on infection was drawn, showing the marginal effect relationship between important characteristics and outcome variables (how important influencing factors affect retinopathy). TyG> 4 showed significant impact (**Figure 6**). Multicollinearity assessment showed all VIF values were below 1.4 for both machine learning and logistic regression models, well under the threshold of 5, indicating no significant multicollinearity concerns.

## 4 Discussion

Early screening and identification of people at high risk of developing DR is important for prevention and treatment. However, Li et al. (39) showed that only 17.48% of T2DM undergo routine DR Screening yearly. Doctors in the diabetes department, especially in primary hospitals that lack equipments and professionals, often overlook the early signs of retinopathy in their patients (36, 37). This study introduces a paradigm shift in diabetic retinopathy (DR) risk stratification by integrating the triglyceride-glucose index (TyG) – an easily measurable, low-cost surrogate of insulin resistance (IR) – into machine learning prediction models. Unlike prior models that either overlook IR (40–42) or rely on impractical biomarkers like HOMA-IR (requiring specialized insulin assays) (11), our approach leverages routinely available lipid/glucose data, addressing a critical barrier in primary care settings. The prominent contribution of TyG in SHAP analysis (**Figures 4**–**6**) further establishes its role as a novel, interpretable predictor for scalable DR screening.

Results showed that logistic regression and SVM had the best performance among the five models, with area under ROC curve of the testing set and training set of 0.85 and 0.82 respectively. Similarly, Tsao et al. (43) showed that the SVM model has good predictive performance. Jiang et al. (37) also showed that logistic regression has good predictive performance. LR is a linear model suitable for small sample analysis, and it is sensitive to outliers. SVM finds the optimal hyperplane by optimizing the objective function, which is suitable for the analysis of complex data (37). Different algorithms have their own advantages in the modeling process, and no algorithm can be applied to all models. Therefore, the corresponding model should be selected according to the research design. This study was conducted based on the computational minimum sample size. Therefore, a study with a larger sample size is needed to compare the performance between LR and SVM. Our SVM model demonstrated test set AUC (0.85) comparable to Jiang et al. (37) (AUC = 0.89) and superior to Wang et al. (40) (AUC = 0.709). The integration of TyG provided predictive power comparable to HOMA-IR-based models (11) without requiring specialized tests. Unlike Roşu et al. (42) (AUC = 0.72) and Yang et al. (32) (AUC = 0.78) which excluded insulin resistance indicators, our TyG-incorporated model achieved higher AUC (0.85) with clinically accessible variables. This balances performance and feasibility for primary care settings.While our initial hold-out test set showed excellent performance (AUC >0.83), the additional 5-fold cross-validation provides stronger evidence of model robustness. The cross-validated AUC remained above 0.81 with standard deviations <0.02, confirming reliable performance across diverse data partitions.

Herein, the variables included in LR and SVM were consistent. The remaining variables were described in previous studies except for the significant predictive ability of TyG (9, 31, 37, 44, 45). Previous studies showed that the interpretability of ML models is challenging (46). In the present study, SHAP was used to improve model interpretability. SHAP is used to interpret a "black box" model that calculates a Shapley value for each feature in the prediction model to assess the importance of all feature combinations, reflecting their contribution to the predictive power of the overall model (26). SHAP results showed that the top 5 variables included TyG, HDL, Insulin therapy, Diabetes course, and HbA1c.

This study observed discordant results for Smoking and LDL between univariate and multivariate analyses, with a notable reversal in smoking's effect direction (univariate: lower prevalence in DR group, *P* = 0.007; multivariate: protective association, OR = 0.759, *P* = 0.008). This reversal is explained by two synergistic mechanisms: First, hypertension mediation—hypertension was more prevalent in the DR group (56.25% vs 44.32%, *P* < 0.001) and strongly predicted DR (OR = 1.506, *P* < 0.001); since smoking promotes hypertension (47), adjusting for it in multivariate models revealed smoking's residual protective effect *via* metabolic pathways. Second, exclusion of severe comorbidities may favor resilient smokers, consistent with the "smoker’s paradox" in chronic disease cohorts (48, 49). Regarding LDL and HDL, their reversals reflect collinearity within lipid metabolism (univariate analysis failed to disentangle HDL/LDL effects from TyG, which correlates with triglycerides) and a synergistic risk interaction where low HDL combined with high TyG increases DR risk—captured only by multivariate models. These reversals underscore that risk factors' net effects depend on comorbidities: Heavy smokers with hypertension remain high-risk despite smoking's statistical "protection," while LDL's reversal highlights complex lipid interactions in DR pathogenesis.

TyG emerged as a key predictor with dual significance. Notably, increased TyG levels were associated with a high risk of DR due to the excessive production of mitochondrial superoxide in microvascular endothelial cells. This production is caused by pathway-specific insulin resistance, meanwhile, triggering intracellular hyperglycemia and vascular damage (50). As a biochemical indicator reflecting synergistic dysregulation of glucose/lipid metabolism (51), TyG directly links to DR pathogenesis. Our SHAP analysis revealed a “non-linear risk surge” at TyG>4, aligning with the point where dysregulated glucose/lipid metabolism triggers mitochondrial superoxide overproduction in retinal microvessels – a key driver of early vascular damage. Based on multivariate regression, each unit increase in TyG elevates DR risk by 9.1% (OR = 1.091, 95%CI:1.005–1.185). The threshold effect at TyG>4 reflects amplified risk accumulation consistent with Zhou et al.'s (52) report. This threshold is calculable from routine lipid/glucose tests. We recommend annual fundus screening for patients exceeding TyG>4. Crucially, TyG overcomes limitations of traditional IR metrics. Our study is the first to operationalize TyG within a predictive algorithm, with its threshold effect (TyG>4) providing actionable clinical guidance.Though TyG cut-offs vary by disease context [e.g., 4.65 for IR (16), 8.88 for diabetes progression (53)], our threshold specifically targets DR risk stratification in primary care settings where standardized values remain undefined. A meta-analysis (54) showed that there is no standardized threshold value for TyG and clear threshold value related to TyG and DR. Therefore, more high-quality studies are needed to determine the optimal cut-off point for TyG in the future.

Insulin therapy is another key predictor of DR. Wang, Li et al (40, 45) found that the insulin treatment is associated with a high risk of DR development, possibly because insulin therapy indicates worse islet function. While Ricard found it possibly related to the rapid reduction of blood glucose (55). Nonetheless, more basic research is needed to confirm the findings. This study, along with others, has demonstrated that the duration of diabetes mellitus is closely associated with the development of DR (30, 41, 44, 56), possibly due to the prolonged exposure of blood vessels to risk factors.

Herein, DR was linked to increased HBA1c levels. This may be due to the continuous elevation of blood glucose, which can lead to dysfunction of the retinal vascular endothelium and cause retinal ischemia and increased vascular permeability (2). Similar findings have been reported previously (11, 30, 45).

In addition, HDL was identified as a key predictor of DR. Low HDL levels may indicate a higher risk of DR, consistent with Roşu, Liu, Li et al (42, 57, 58).

By addressing three critical gaps – 1) IR assessment simplification *via* TyG, 2) cross-tier validation (urban tertiary/rural primary hospitals), and 3) SHAP-driven interpretability – our model enables scalable DR screening. Our model requires only 9 routinely collected variables, enabling seamless integration into electronic health records (EHR) for real-time risk stratification during diabetic outpatient visits. Based on SHAP probability thresholds (**Figures 4**-**6**), we propose a tiered screening pathway: High-risk patients (TyG >4 and other key predictors elevated): Prioritize immediate ophthalmologist referral. Low-to-intermediate-risk patients: Maintain routine annual/biannual retinal screening. This approach is particularly valuable for resource-limited settings (e.g., rural clinics), where the model can optimize specialist resource allocation by focusing on high-risk individuals identified through universally available tests like TyG (calculated from routine lipid/glucose tests). Besides, Our model exhibits inherent generalizability potential through its reliance on universal predictors requiring only routine clinical data, demonstrated demographic resilience across diverse populations, and established scalability pathways including a simplified screening protocol and ongoing multi-region validation.

This study bridges a translational gap: while TyG-DR associations were reported, we are the first to validate its clinical utility within a predictive algorithm. The integration with SHAP interpretation further distinguishes our model from conventional approaches. while retrospective data from two hospitals with different resource levels (urban tertiary vs. rural primary) may introduce selection bias, this heterogeneity enhances real-world generalizability.

Limitation: First, only internal verification was conducted, thus effective external verification is needed. Secondly, excluding high-missingness variables may omit useful information, and while mean/mode imputation is practical, advanced techniques (e.g., multiple imputation) might reduce potential bias. Last, this study did not differentiate early vs. late-stage DR in predictions due to ungraded diagnostic records. However, our model prioritizes biomarkers linked to DR progression (TyG, diabetes course). Future work should incorporate standardized severity staging to enable stage-specific optimization.

## 5 Conclusion and recommendations

In this study, a DR prediction model was built using TyG and other easily available clinical data. LR and SVM models had the best performance. SHAP showed that the most important predictors of DR were TyG, insulin therapy, diabetes course, HbA1c, and HDL. These findings suggest that baseline characteristics of patients can be used to screen for high-risk DR to improve early diagnosis and implement timely referral for patients.

## Data Availability

The raw data supporting the conclusions of this article will be made available by the authors, without undue reservation.
