# Supplementary figures and images for: Development of machine learning predictive model for type 2 diabetic retinopathy using the triglyceride-glucose index explained by SHAP method
Source: Front Endocrinol (Lausanne). 2025 Nov 10;16:1631647. doi: 10.3389/fendo.2025.1631647 (PMC12640817; doi:10.3389/fendo.2025.1631647)

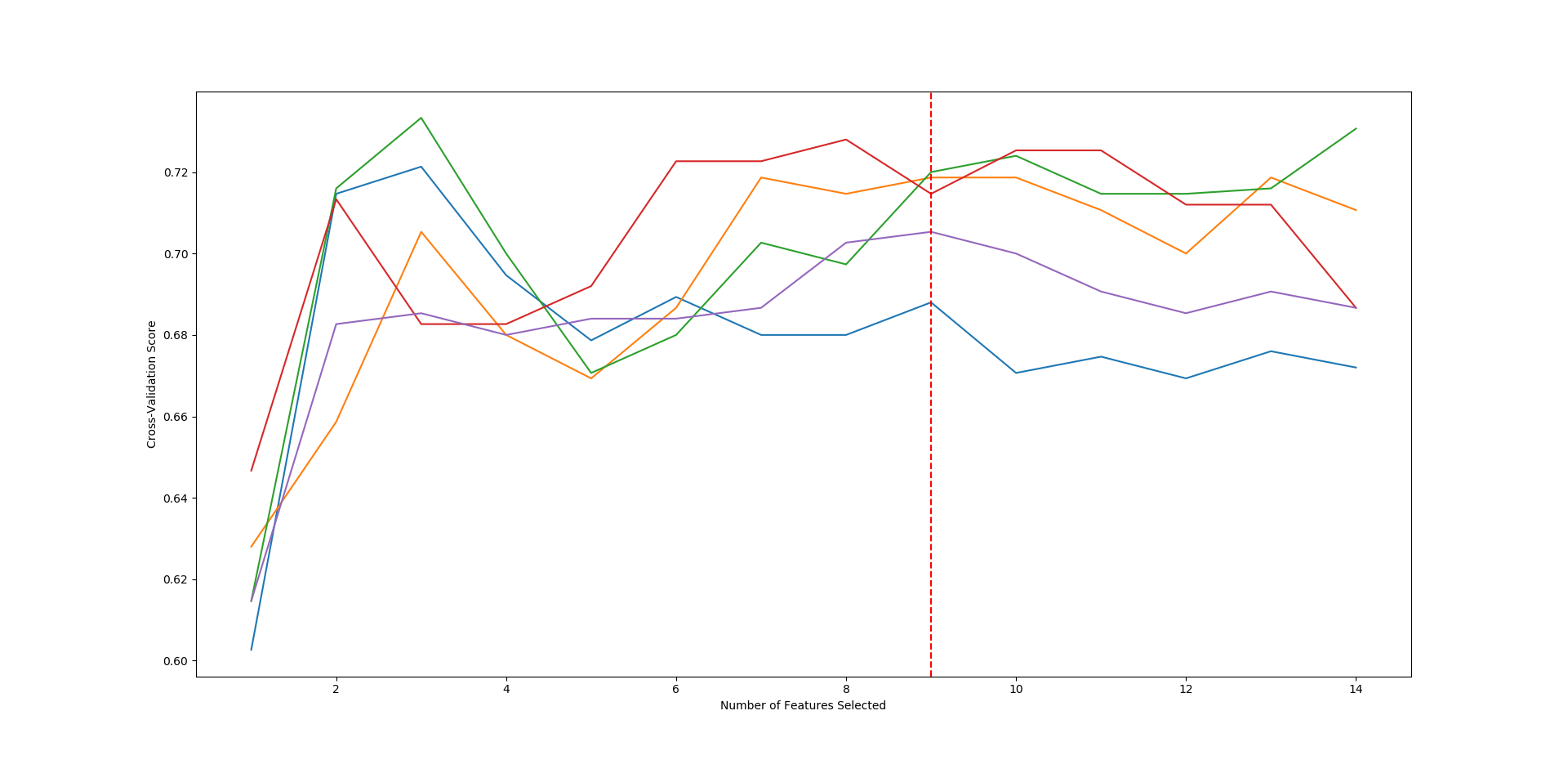

Supplement: Supplementary file 1 [file Image1.png]

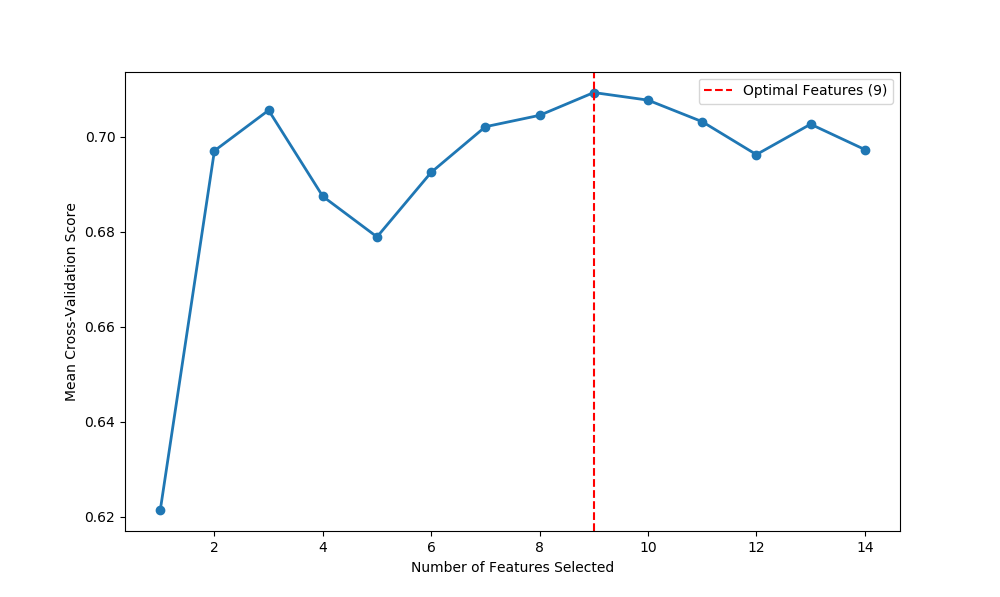

Supplement: Supplementary file 2 [file Image2.png]
